# ω-6 Polyunsaturated fatty acids (linoleic acid) activate both autophagy and antioxidation in a synergistic feedback loop via TOR-dependent and TOR-independent signaling pathways

**DOI:** 10.1038/s41419-020-02750-0

**Published:** 2020-07-30

**Authors:** Bo Yang, Yan Zhou, Mengjiao Wu, Xueshan Li, Kangsen Mai, Qinghui Ai

**Affiliations:** 1 Key Laboratory of Aquaculture Nutrition and Feed (Ministry of Agriculture and Rural Affairs) & Key Laboratory of Mariculture (Ministry of Education), Ocean University of China, 5 Yushan Road, 266003 Qingdao, Shandong People’s Republic of China; 2 Laboratory for Marine Fisheries Science and Food Production Processes, Qingdao National Laboratory for Marine Science and Technology, 1 Wenhai Road, 266237 Qingdao, Shandong People’s Republic of China

**Keywords:** Macroautophagy, Nutrient signalling, Stress signalling

## Abstract

ω-6 Polyunsaturated fatty acids (PUFAs) are essential fatty acids that participate in macroautophagy (hereafter referred to as autophagy) and the Kelch ECH-associating protein 1 (Keap1)—nuclear factor erythroid 2-related factor 2 (Nrf2) antioxidant system in organisms. However, the molecular mechanisms by which ω-6 PUFAs (linoleic acid) regulate autophagy and Keap1–Nrf2 antioxidant system are not completely understood. Therefore, the purposes of this study were to explore the molecular mechanisms by which ω-6 PUFAs (linoleic acid) regulate autophagy and antioxidant system and to investigate the potential relationship between autophagy and antioxidant system through transcriptomic analysis, quantitative real-time polymerase chain reaction (RT-qPCR), western blot analysis, coimmunoprecipitation (Co-IP) and electrophoretic mobility shift assays (EMSAs) in vivo and in vitro. The results of the present study indicated that ω-6 PUFAs in diets induced autophagy but decrease antioxidant ability in vivo. However, the results also provided evidence, for the first time, that ω-6 PUFAs (linoleic acid) induced autophagy and increased antioxidant ability through the adenosine monophosphate-activated protein kinase (AMPK) signaling pathway and the AMPK-target of rapamycin (TOR) signaling pathway in hepatocytes in vitro. Interestingly, the findings revealed a ω-6 PUFA-induced synergistic feedback loop between autophagy and antioxidant system, which are connected with each other through the P62 and Keap1 complex. These results suggested that ω-6 PUFAs (linoleic acid) could be useful for activating a synergistic feedback loop between autophagy and antioxidant system and could greatly aid in the prevention and treatment of multiple pathologies.

## Introduction

ω-6 Polyunsaturated fatty acids (PUFAs) are essential fatty acids that participate in multiple types of cellular metabolism^1^. A considerable number of studies have demonstrated the antistress activity mediated by ω-6 PUFAs, including alleviation of oxidative stress, modulation of cyclooxygenase activity and changes in membrane phospholipid composition and receptor function^2–5^. However, the stress-preventive mechanisms mobilized by ω-6 PUFAs are not completely understood.

Two such host defense mechanisms, autophagy and Keap1–Nrf2 system-mediated antioxidation, are associated with metabolic pathways and innate immunity, and dysregulation of these processes is associated with the pathogenic mechanisms of multiple human diseases^6–9^. Previously, several reports have suggested that autophagy and antioxidation are altered in sensitive cancer cell lines and that these mechanisms display cytotoxic and/or cytostatic responses to physiological doses of PUFAs^10,11^. However, the molecular mechanisms by which ω-6 PUFAs regulate autophagy and antioxidation has not been reported. Recently, several studies have shown that PUFAs are positive inducers of AMPK and TOR signaling pathways^12^. AMPK has been identified as a novel inducer of Nrf2 in U251 cells that functions via ATP-depletion-induced AMPK activation and consequent mTOR inhibition^13^. In addition, a previous study focused on the regulatory effects of ω-3 PUFAs on autophagy via the AMPK–TOR signaling pathway^14^. However, whether the AMPK and TOR signaling pathways participate in autophagy and antioxidation induced by ω-6 PUFAs is not completely understood.

The autophagy and antioxidation signaling pathways are complex and seem to be interrelated under antioxidant-supplemented conditions. In conditional Atg7 knockout mice, it has been reported that inhibition of autophagy in the liver causes P62 to accumulate and strongly induces phase II drug-metabolizing enzymes and antioxidant proteins^15^. In mice model of oxidative stress caused by ischemia/reperfusion, supplementation of vitamin D has been found to improve antioxidant capacity by regulating autophagy and relieving oxidative stress^16^. Most antioxidants induce autophagy and antioxidation by regulating the expression of the P62–Keap1–Nrf2 signaling pathways, thereby relieving oxidative stress and reducing apoptosis. Recent studies have confirmed that a marine ω-3 PUFA (docosahexaenoic acid, DHA) evokes cytoprotection against oxidative stress and protein misfolding by inducing both autophagy and Nrf2 in human retinal pigment epithelial cells^17^. However, whether there is a potential relationship between autophagy and antioxidant system mediated by ω-6 PUFAs through the P62 and Keap1 complex has not been elucidated.

Keap1–Nrf2-mediated antioxidation and autophagy, are involved in the pathogenic mechanisms of multiple human diseases. Although fish are relatively lower vertebrates than mammals from an evolutionary perspective, they have immune defense systems similar to mammals, making them perfect and often-used model animals for research on the pathogenesis of human diseases. In our laboratory, Tan et al.^18^ found that ω-6 PUFAs (linoleic acid) influence the antioxidant system of large yellow croaker (*Larimichthys crocea*), a commercially important fish species in China. However, the specific molecular mechanisms remain unknown. Thus, this study aimed to investigate how ω-6 PUFAs (linoleic acid) regulate autophagy and antioxidation and whether there is a potential relationship between these two ω-6 PUFA-mediated mechanisms. Our findings might facilitate the discovery of new approaches to improve immune function in multiple human diseases.

## Results

### Dietary ω-6 PUFA supplementation induces autophagy but decreases antioxidant ability in vivo

**Fig. 1:**
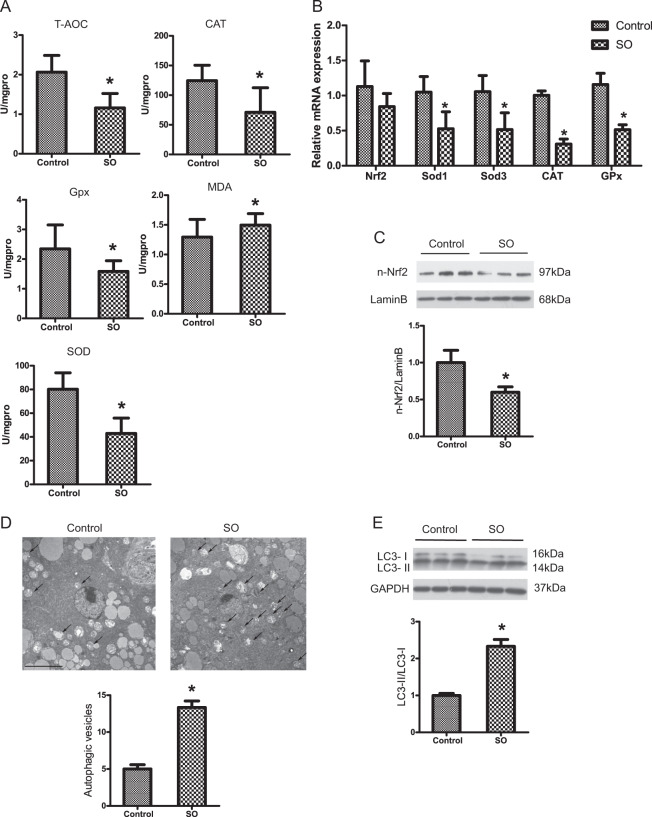
Dietary ω-6 PUFA supplementation induces autophagy but decreases antioxidant ability in vivo. Activity of antioxidant enzyme (CAT, SOD, MDA, Gpx, and T-AOC) were examined in the livers of experimental fish (*n* = 3) (**a**). The mRNA expression levels of key hepatic antioxidation-related genes (Nrf2, Sod1, Sod3, CAT, and Gpx) were analyzed (*n* = 6) (**b**). Nuclear Nrf2 in the liver was examined by western blot analysis and quantitated after treatment with different diets (*n* = 3) (**c**). Macroautophagy in the liver was identified by TEM after treatment with different diets. Scale bars, 5 μm (**d**). The LC3-II/LC3-I ratio in the liver was examined by western blot analysis and quantitated after treatment with different diets (*n* = 3) (**e**). The FO diet group was the control group. The results are presented as the mean with SEM and were analyzed using independent *t*-tests (*P < 0.05).

Omics technologies enable global and in-depth characterization of physiological reactions produced by stimuli^19^. We used transcriptomics to investigate the effects of ω-6 PUFAs on autophagy and antioxidation in vivo. A total of 1237 differentially expressed genes were detected, including 580 upregulated genes and 657 downregulated genes (Supplementary Fig. 1A). According to the GO terms, 2437 genes were classified into three major functional categories: 1380 genes in the biological process category, 356 genes in the cellular component category, and 701 genes in the molecular function category (Supplementary Fig. 1B). To identify the active biological pathways in large yellow croaker, 1237 differentially expressed genes were mapped to canonical signaling pathways found in the Kyoto Encyclopedia of Genes and Genomes (KEGG) database (Supplementary Fig. 1C). Ultimately, a total of 803 genes were mapped to 9 statistically significant categories (*P* < 0.05). Among these pathways, metabolic pathways were represented by 64 downregulated genes. Ten upregulated genes and five downregulated genes were involved in the PPAR signaling pathway (Supplementary Fig. 2A). KEGG analysis further identified numerous unigenes that were enriched in various known immunity-related pathways, such as the peroxisome pathway, the p53 signaling pathway, the mTOR signaling pathway and the phagosome pathway (Supplementary Fig. 2B). On the basis of the transcriptomic evidence, we examined the effects of ω-6 PUFAs on autophagy and antioxidation. The supplementation of SO decreased the activity of the antioxidant enzymes catalase (CAT), superoxide dismutase (SOD), and glutathione peroxidase (Gpx); decreased total antioxidant capacity (T-AOC); and increased the content of malondialdehyde (MDA) (Fig. 1a). In addition, the mRNA expression levels of Nrf2, SOD1, SOD3, CAT, and Gpx in the fish fed the SO diet were significantly lower than those in the fish fed the FO diet (Fig. 1b). Furthermore, the protein levels of nuclear Nrf2, a biomarker for antioxidation, were decreased in the SO group compared to the FO group (Fig. 1c). Autophagy has been shown to be a cellular antioxidative process that helps alleviate lipid-induced oxidative stress and regulate antioxidation^17^. Therefore, we determined whether dietary ω-6 PUFAs activate autophagy. The transcriptome results revealed that the transcriptional levels of autophagy-related genes were not significantly different between the FO and SO groups (Supplementary Fig. 2C). However, electron microscopy (EM) observations demonstrated that the supplementation of SO increased autophagosome formation (Fig. 1d). We assessed the protein levels of the autophagosome marker microtubule-associated protein 1 light chain 3 (LC3) by immunoblot analysis and found that SO upregulated the LC3-II/LC3-I protein expression ratio (Fig. 1e). Thus, dietary ω-6 PUFA supplementation induces autophagy but decreases antioxidant ability in vivo.

### ω-6 PUFAs induce autophagy and increase antioxidant ability in hepatocytes in vitro

**Fig. 2:**
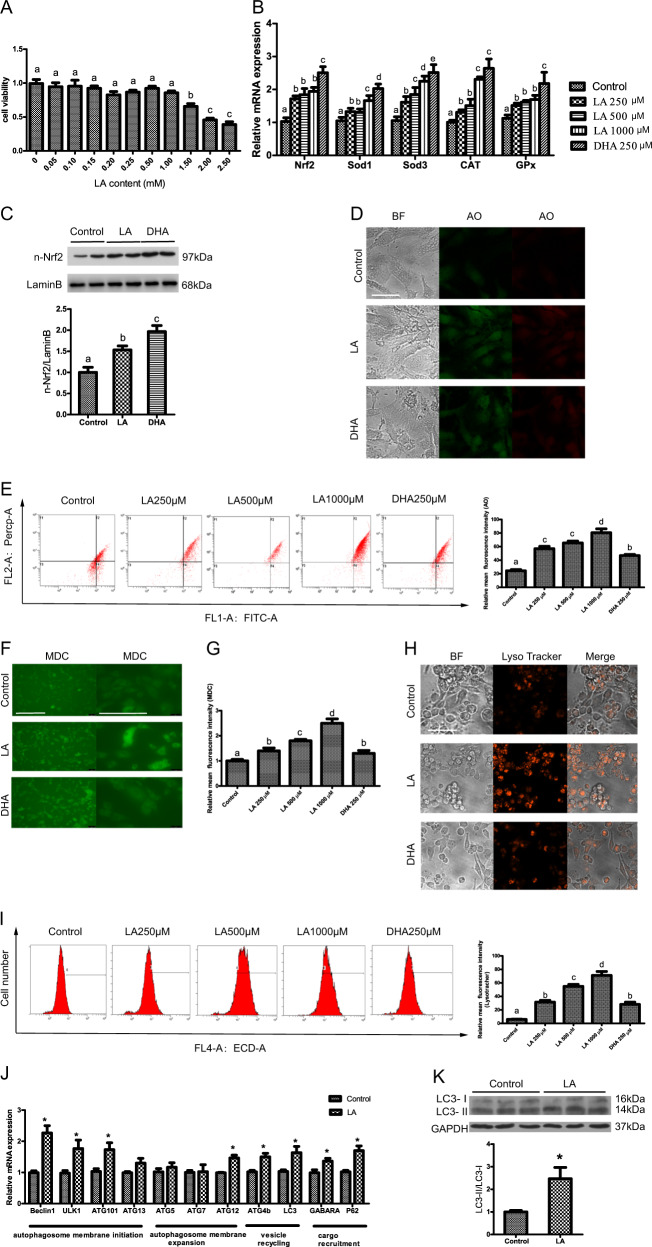
ω-6 PUFAs induce autophagy and increase antioxidant ability in hepatocytes in vitro. Serum-starved cells were treated with the indicated concentrations of LA or DHA for 24 h. The viability of the hepatocytes was evaluated with a CCK8 Assay Kit after LA treatment (*n* = 3) (**a**). The mRNA expression levels of key antioxidation-related genes (Nrf2, Sod1, Sod3, CAT, and Gpx) were analyzed (*n* = 6) (**b**). Nuclear Nrf2 was examined by western blot analysis and quantitated after treatment with LA or DHA (*n* = 3) (**c**). Representative confocal microscopic image of hepatocytes stained with AO. Scale bars, 50 μm (**d**). The presence of AO-stained intracellular autophagic vacuoles was demonstrated by flow cytometry, which revealed a dose-dependent increase in the red:green (FL2:FL1) fluorescence ratio, and the numbers of the AO-stained intracellular autophagic vacuoles were calculated (**e**). Representative confocal microscopic image of hepatocytes stained with MDC. Scale bars, 200 and 100 μm (**f**). The numbers of the MDC-stained intracellular autophagosomes were calculated with a fluorescence microplate assay of blue fluorescence intensity (**g**). Representative confocal microscopic image of hepatocytes stained with LysoTracker. Scale bars, 50 μm (**h**). The presence of LysoTracker-stained intracellular autolysosomes was demonstrated by flow cytometry, which revealed a dose-dependent increase in red (FL4) fluorescence intensity, and the numbers of the LysoTracker-stained intracellular autolysosomes were calculated by flow cytometric analysis of the mean red (FL4) fluorescence intensity (**i**). The mRNA expression levels of key autophagy-related genes (Beclin1, ULK1, ATG101, ATG13, ATG5, ATG7, ATG12, ATG4b, LC3, GABARA, and P62) were analyzed (*n* = 6) (**j**). The LC3-II/LC3-I ratio was examined by western blot analysis and quantitated after treatment with LA (*n* = 3) (**k**). The results are presented as the mean with SEM and were analyzed using independent *t-*tests and Tukey’s test. Bars bearing the same letters are not significantly different among treatments (*P ≥ 0.05).

To gain insight into the mechanisms of the effects of ω-6 PUFAs on autophagy and antioxidation, several in vitro experiments have been conducted on hepatocytes from large yellow croaker. FO is rich in ω-3 PUFAs such as DHA, and SO is rich in ω-6 PUFAs such as linoleic acid (LA). Therefore, in vitro, dietary FO supplementation was represented by DHA treatment, and dietary SO supplementation was represented by LA treatment. Serum-starved cells were treated with 2% BSA as a control. A Cell Counting Kit-8 (CCK8) assay showed that LA concentrations up to 1 mM had no influence on the viability of hepatocytes (Fig. 2a). Compared with the BSA treatment group, the mRNA expression levels of Nrf2, SOD1, SOD3, CAT, and Gpx were significantly increased in the LA groups with increasing LA levels from 250 to 1000 μM. However, the mRNA expression levels in the LA groups were lower than those in the DHA group (Fig. 2b). The protein levels of nuclear Nrf2 increased after 250 μM LA and DHA treatment (Fig. 2c). Next, we used five different methods to assess autophagosome formation. Depending on the acidity, acridine orange (AO) caused autophagic lysosomes to appear as orange/red fluorescent vesicles, while it caused nuclei to appear green. AO staining demonstrated that LA dose-dependently increased the numbers of intracellular acidic compartments (Fig. 2d, e). Monodansylcadaverine (MDC), a specific in vitro marker for autophagic vesicles, was also used. MDC staining demonstrated that LA increased the numbers of autophagosomes (green dots) (200× and 630×) (Fig. 2f, g). LysoTracker Red was further used to assess lysosomal activity and autolysosome function. LysoTracker staining indicated that autophagic flux increased after LA incubation (Fig. 2h, i). The acidic intracellular vesicles were visualized by MDC, AO, and LysoTracker staining, which revealed that the LA group exhibited greater autophagic activity than the DHA group. To determine how LA modulated autophagosome formation, key hepatic autophagy-related genes (associated with autophagosome membrane initiation and expansion, vesicle recycling, and cargo recruitment) were measured. The mRNA expression levels of the autophagy-related genes Beclin1, ULK1, autophagy-related 101 (ATG101), autophagy-related 12 (ATG12), autophagy-related 4B (ATG4B), LC3, GABARA, and P62 were higher in the LA group than in the control group (Fig. 2j). The LC3-II/LC3-I ratio also significantly increased after LA treatment (Fig. 2k). Thus, ω-6 PUFAs increase antioxidant ability and induce autophagy in hepatocytes in vitro.

### ω-6 PUFAs activate autophagy and antioxidation through the AMPK signaling pathway

**Fig. 3:**
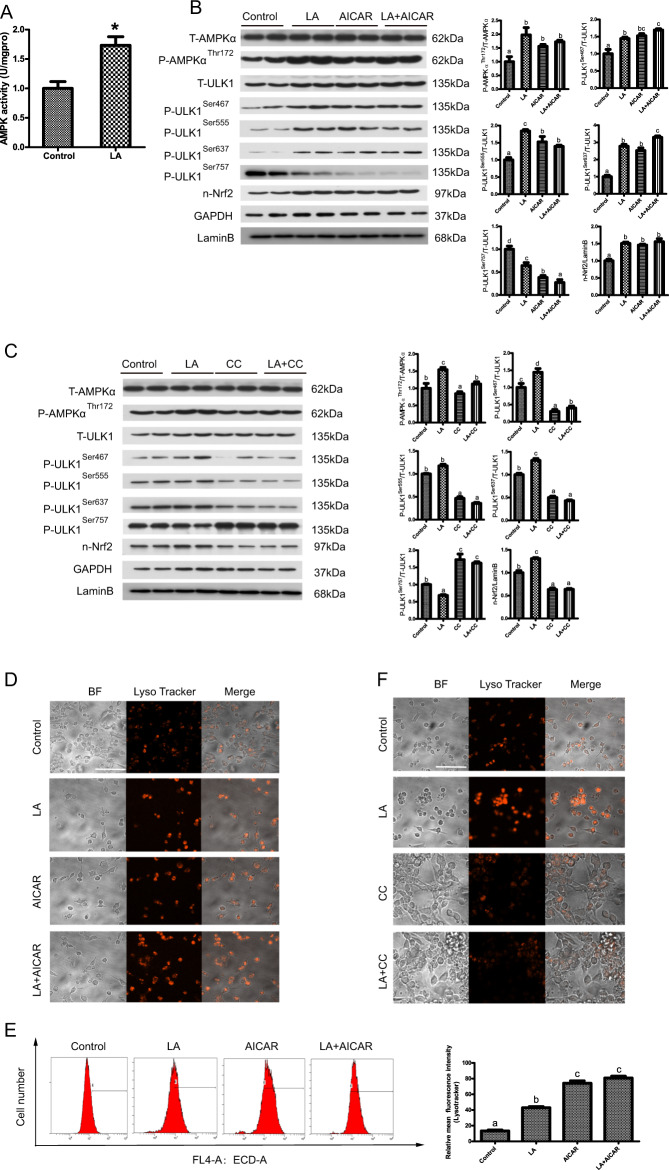

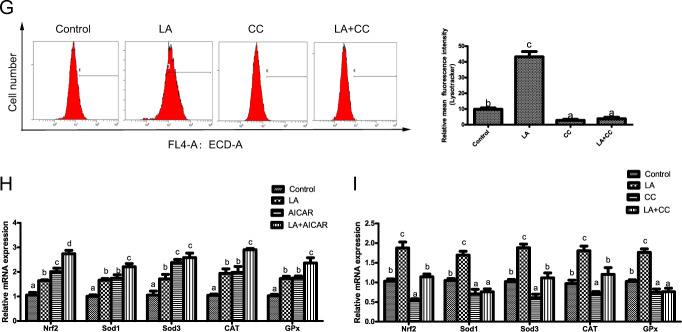
ω-6 PUFAs activate autophagy and antioxidation through the AMPK signaling pathway. AMPK activity was evaluated in hepatocytes incubated for 24 h in the control or 250 μM LA-containing medium (*n* = 3) (**a**). The levels of total and phosphorylated AMPKα and ULK1 and the levels of nuclear Nrf2 were examined by western blot analysis and quantitated after treatment with an AMPK pathway inhibitor (5 μM CC) or activator (500 μM AICAR) (*n* = 3). 10 gels were run and 10 blots were made. All of the blots were not stripped and re-probed. The blots of T-AMPKα, P-AMPKα^Thr172^, T-ULK1, P-ULK1^Ser467^, P-ULK1^Ser555^, P-ULK1^Ser637^, and P-ULK1^Ser757^ were used for the GAPDH loading controls and the blots of n-Nrf2 was used for the LaminB loading controls (**b**, **c**). Representative confocal microscopic image of hepatocytes stained with LysoTracker after treatment with an AMPK pathway inhibitor or activator. Scale bars, 50 μm (**d**, **f**). The presence of LysoTracker-stained intracellular autolysosomes was demonstrated by flow cytometry, and the numbers of the autolysosomes were calculated by flow cytometric analysis of the mean red (FL4) fluorescence intensity after treatment with an AMPK pathway inhibitor or activator (**e**, **g**). The mRNA expression levels of key antioxidation-related genes (Nrf2, Sod1, Sod3, CAT, and Gpx) were analyzed after treatment with a pathway inhibitor or activator (*n* = 6) (**h**, **i**). The results are presented as the mean with SEM and were analyzed using independent *t*-tests and Tukey’s test. Bars bearing the same letters are not significantly different among treatments (*P ≥ 0.05).

Having obtained convincing evidence that LA could activate autophagy and antioxidation, we next investigated the possible mechanism by which LA exerted these effects in hepatocytes. The AMPK pathway mediates important regulatory actions on both autophagy and antioxidation^20^. LA upregulated AMPK activity, indicating that it activated signaling pathways involving AMPK (Fig. 3a). To determine the relative contributions of AMPK pathways to the activation of autophagy and antioxidation in response to LA, we used 5-aminoimidazole-4-carboxamide ribonucleotide (AICAR) and Compound C (CC), which activate and inhibit AMPK, respectively. Compared with the control group, the LA group exhibited increased phosphorylation of AMPKα (Thr172) and ULK1 (Ser467, Ser555, and Ser637) and increased levels of nuclear Nrf2 but decreased phosphorylation of ULK1 (Ser757) (Fig. 3b). The results also indicated that the phosphorylation of AMPKα (Thr172) and ULK1 (Ser467, Ser555, and Ser637) and the levels of nuclear Nrf2 were increased, while the phosphorylation of ULK1 (Ser757) was decreased, in the LA+AICAR group (Fig. 3b). CC pretreatment before LA treatment (in the LA+CC group) decreased the phosphorylation of AMPKα (Thr172) and ULK1 (Ser467, Ser555, and Ser637) and the levels of nuclear Nrf2 but increased the phosphorylation of ULK1 (Ser757) (Fig. 3c). Furthermore, autophagic flux was quantified by flow cytometry with LysoTracker staining. The results indicated that the mean red fluorescence intensity increased after LA+AICAR incubation (Fig. 3d, e). CC pretreatment (in the LA+CC group) alleviated the LA-induced increase in autophagic flux (Fig. 3f, g). In addition, the mRNA expression levels of Nrf2, SOD1, SOD3, CAT, and Gpx were significantly increased after LA+AICAR treatment (Fig. 3h). In contrast, the mRNA expression levels of these genes were significantly decreased after LA+CC treatment (Fig. 3i). These results indicated that ω-6 PUFAs activate autophagy and antioxidation through the AMPK signaling pathway.

### ω-6 PUFAs activate autophagy and antioxidation via TOR-dependent and TOR-independent signaling pathways

**Fig. 4:**
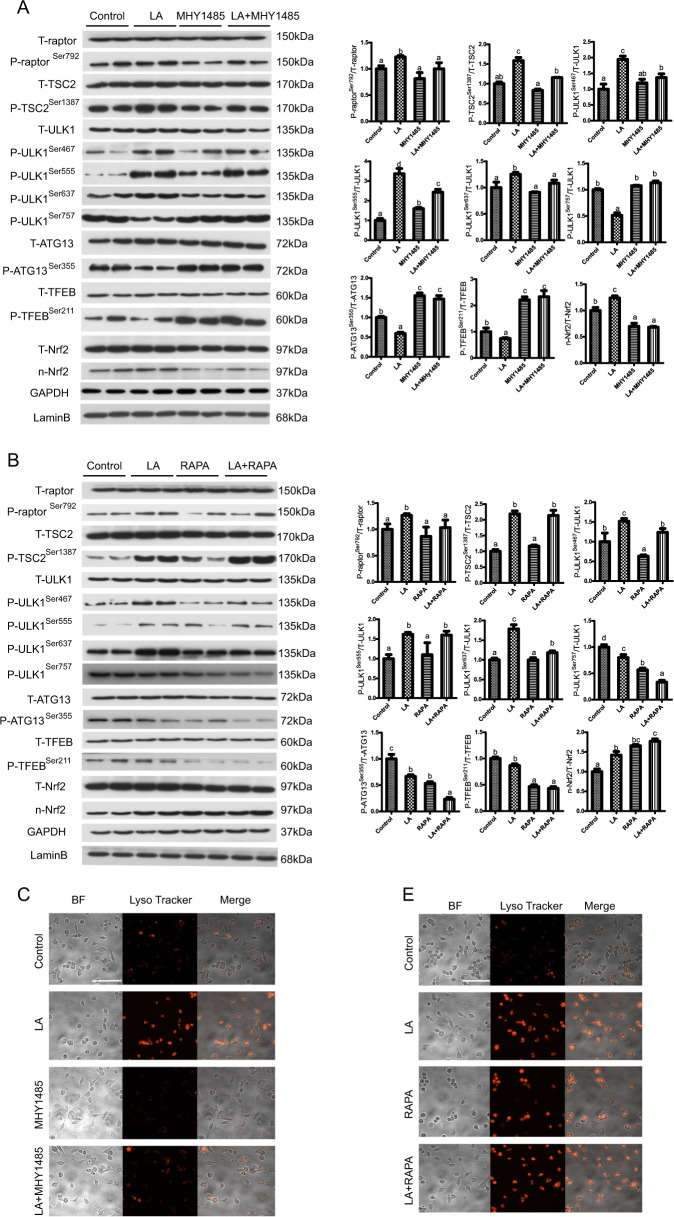

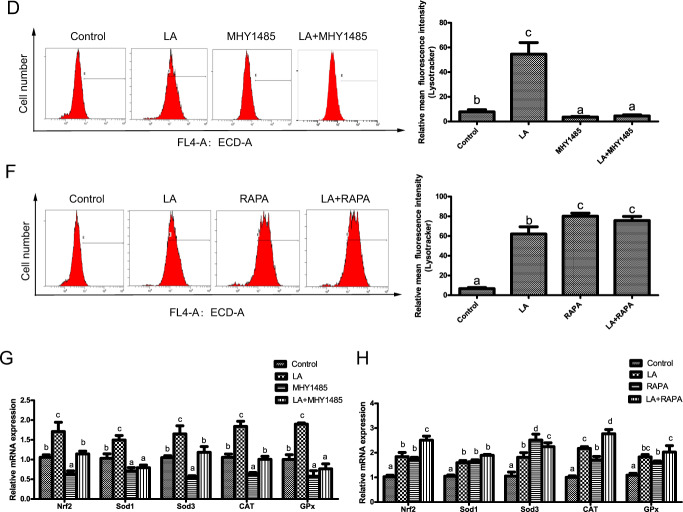
ω-6 PUFAs activate autophagy and antioxidation via TOR-dependent and TOR-independent signaling pathways. The levels of total and phosphorylated raptor, TSC2, ULK1, ATG13, and TFEB and the levels of nuclear Nrf2 were examined by western blot analysis and quantitated after treatment with a TOR pathway inhibitor (500 nM RAPA) or activator (2 μM MHY1485) (*n* = 3). 17 gels were run and 17 blots were made. All of the blots were not stripped and re-probed. The blots of T-raptor, P-raptor^Ser792^, T-TSC2, P-TSC2^Ser1387^, T-ULK1, P-ULK1^Ser467^, P-ULK1^Ser555^, P-ULK1^Ser637^, P-ULK1^Ser757^, T-ATG13, P-ATG13^Ser355^, T-TFEB, P-TFEB^Ser211^, and T-Nrf2 were used for the GAPDH loading controls and the blots of n-Nrf2 was used for the LaminB loading controls (**a**, **b**). Representative confocal microscopic image of hepatocytes stained with LysoTracker after treatment with a TOR pathway inhibitor or activator. Scale bars, 50 μm. The same type of samples (LA group) was used in TOR pathway inhibitor (RAPA) group and autophagy inhibitor (3-MA) group (**c**, **e**). The presence of LysoTracker-stained intracellular autolysosomes was demonstrated by flow cytometry, and the numbers of the autolysosomes were calculated by flow cytometric analysis of the mean red (FL4) fluorescence intensity after treatment with a TOR pathway inhibitor or activator (**d**, **f**). The mRNA expression levels of key antioxidation-related genes (Nrf2, Sod1, Sod3, CAT, and Gpx) were analyzed after treatment with a TOR pathway inhibitor or activator (*n* = 6) (**g**, **h**). The results are presented as the mean with SEM and were analyzed using Tukey’s test. Bars bearing the same letters are not significantly different among treatments (*P ≥ 0.05).

Activation of AMPK leads to inhibition of mTOR, a strong inhibitor of autophagy^21^. Recent findings have demonstrated the involvement of the TOR complex in the regulation of autophagy and antioxidation^22^. To determine whether TOR mediates the above-presented effects of LA treatment on autophagy and antioxidation, we used MHY1485 and rapamycin (RAPA), which activate and inhibit TOR, respectively. Compared with the control group, the LA treatment group exhibited increased phosphorylation of raptor (Ser792), TSC2 (Ser1387), and ULK1 (Ser467, Ser555, and Ser637) and increased levels of nuclear Nrf2 but decreased phosphorylation of ULK1 (Ser757), ATG13 (Ser355), and TFEB (Ser211) (Fig. 4a). The results also indicated that the phosphorylation of raptor (Ser792), TSC2 (Ser1387), and ULK1 (Ser467, Ser555, and Ser637) and the levels of nuclear Nrf2 were decreased, while the phosphorylation of ULK1 (Ser757), ATG13 (Ser355), and TFEB (Ser211) was increased, in the LA+MHY1485 group (Fig. 4a). RAPA pretreatment (in the LA+RAPA group) alleviated the LA-induced increases in the phosphorylation levels of raptor (Ser792), ULK1 (Ser467, Ser637, Ser757), ATG13 (Ser355), and TFEB (Ser211) but increased the levels of nuclear Nrf2 (Fig. 4b). Furthermore, autophagic flux was quantified by flow cytometry with LysoTracker staining. The results indicated that the mean red fluorescence intensity increased after LA+RAPA incubation (Fig. 4c, d). MHY1485 pretreatment (in the LA+MHY1485 group) alleviated the LA-induced changes in autophagic flux (Fig. 4e, f). Furthermore, the mRNA expression levels of Nrf2, SOD1, SOD3, CAT, and Gpx were significantly decreased after LA+MHY1485 treatment (Fig. 4g). In contrast, the mRNA expression levels of these genes were significantly increased after LA+RAPA treatment (Fig. 4h). These results indicated that ω-6 PUFAs activate autophagy and antioxidation through TOR-dependent and TOR-independent signaling pathways.

### ω-6 PUFAs increase antioxidant ability via activation of autophagy

**Fig. 5:**
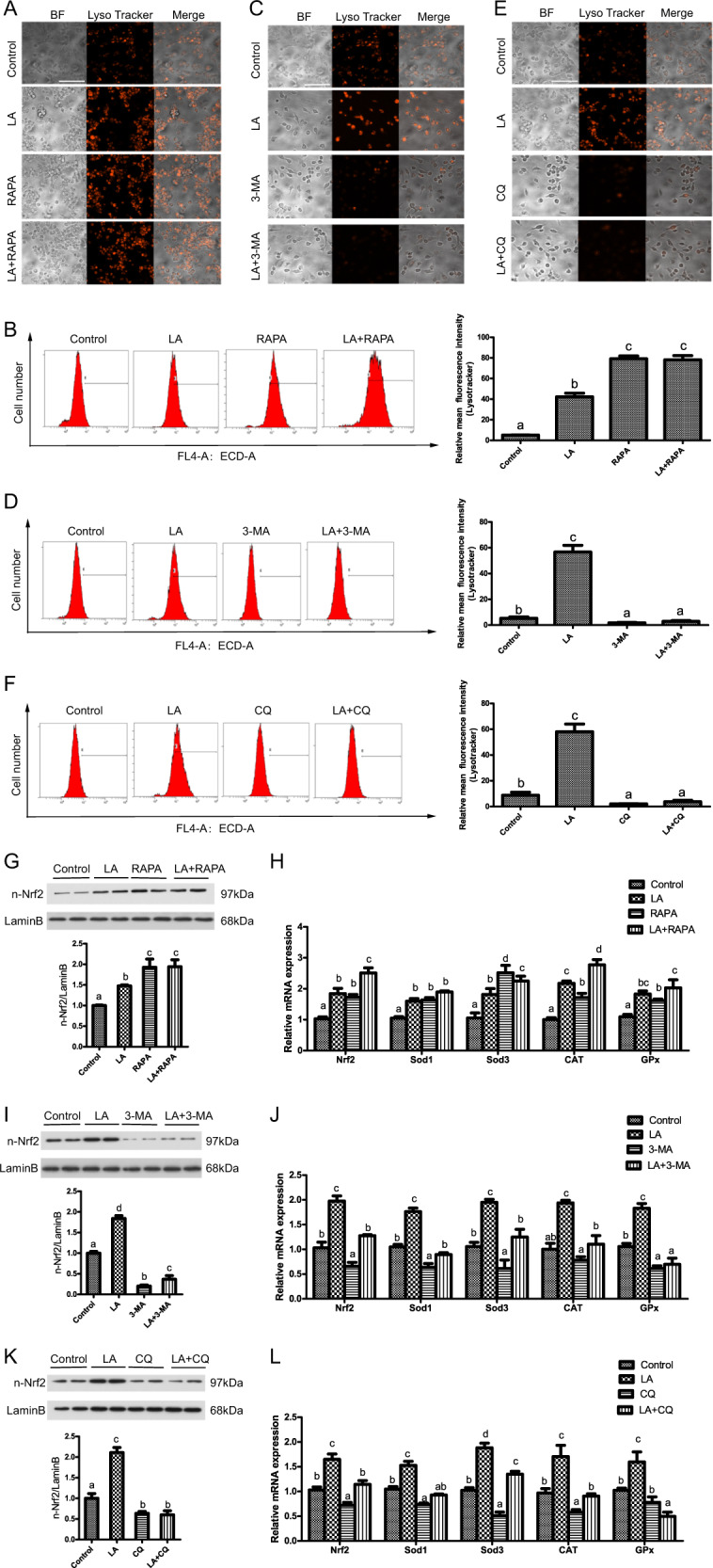
ω-6 PUFAs increase antioxidant ability via activation of autophagy. Representative confocal microscopic image of hepatocytes stained with LysoTracker after treatment with an autophagy inhibitor (5 μM CQ or 2 mM 3-MA) or activator (500 nM RAPA). Scale bars, 50 μm. The same type of samples (LA group) was used in TOR pathway inhibitor (RAPA) group and autophagy inhibitor (3-MA) group (**a**, **c**, **e**). The presence of LysoTracker-stained intracellular autolysosomes was demonstrated by flow cytometry, and the numbers of the autolysosomes were calculated by flow cytometric analysis of the mean red (FL4) fluorescence intensity after treatment with an autophagy inhibitor or activator (**b**, **d**, **f**). Nuclear Nrf2 was examined by western blot analysis and quantitated after treatment with an autophagy inhibitor or activator (*n* = 3) (**g**, **i**, **k**). The mRNA expression levels of key antioxidation-related genes (Nrf2, Sod1, Sod3, CAT, and Gpx) were analyzed after treatment with an autophagy inhibitor or activator (*n* = 6) (**h**, **j**, **l**). The results are presented as the mean with SEM and were analyzed using Tukey’s test. Bars bearing the same letters are not significantly different among treatments (*P ≥ 0.05).

After finding that ω-6 PUFAs could activate autophagy and antioxidation through the AMPK signaling pathway and the AMPK–TOR signaling pathway, we next investigated whether autophagy was directly involved in the LA-induced increase in antioxidant ability. We used the autophagy activator RAPA to promote autophagy and the inhibitors 3-methyladenine (3-MA) and chloroquine (CQ) to block autophagy. The results indicated that LA+RAPA treatment increased the mean red fluorescence intensity (Fig. 5a, b), while LA+3-MA and LA+CQ treatment alleviated the LA-induced increase in autophagic flux (Fig. 5c–f). Consistent with the autophagy levels, the levels of nuclear Nrf2 and the mRNA expression levels of Nrf2, SOD1, SOD3, CAT, and Gpx were significantly increased after LA+RAPA treatment (Fig. 5g, h). In contrast, the levels of nuclear Nrf2 and the mRNA expression levels of Nrf2, SOD1, SOD3, CAT, and Gpx were significantly decreased after LA+3-MA and LA+CQ treatment (Fig. 5i–l). All the above observations indicated that 3-MA and CQ not only decreased LA-induced autophagosome formation but also reduced antioxidant ability, suggesting the involvement of autophagy in the LA-induced increase in antioxidant activity.

### ω-6 PUFAs increase autophagy levels via activation of antioxidant pathways

**Fig. 6:**
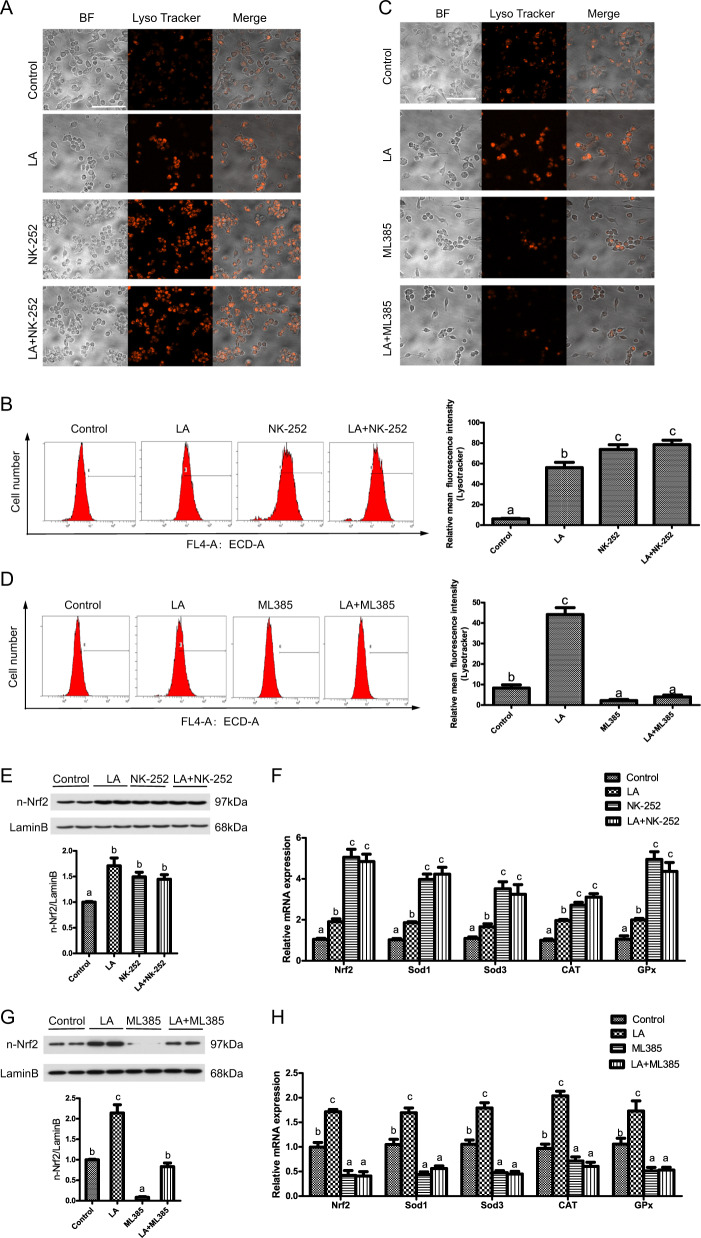
ω-6 PUFAs increase autophagy levels via activation of the antioxidant pathway. Representative confocal microscopic image of hepatocytes stained with LysoTracker after treatment with a Nrf2 pathway inhibitor (5 μM ML385) or activator (2 μM NK-252). Scale bars, 50 μm (**a**, **c**). The presence of LysoTracker-stained intracellular autolysosomes was demonstrated by flow cytometry, and the numbers of the autolysosomes were calculated by flow cytometric analysis of the mean red (FL4) fluorescence intensity after treatment with a Nrf2 pathway inhibitor or activator (**b**, **d**). Nuclear Nrf2 was examined by western blot analysis and quantitated after treatment with a Nrf2 pathway inhibitor (*n* = 3) (**e**, **g**). The mRNA expression levels of key antioxidation-related genes (Nrf2, Sod1, Sod3, CAT, and Gpx) were analyzed after treatment with a Nrf2 pathway inhibitor or activator (*n* = 6) (**f**, **h**). The results are presented as the mean with SEM and were analyzed using Tukey’s test. Bars bearing the same letters are not significantly different among treatments (*P ≥ 0.05).

Having demonstrated that LA increased antioxidant ability via activation of autophagy, we next investigated whether the antioxidant pathway was directly involved in the LA-induced autophagy. Nrf2 is a major regulator of the transcription of antioxidant-related genes^23^. We used NK-252 and ML385 to activate and inhibit Nrf2, respectively. The results indicated that the mean red fluorescence intensity increased after LA+NK-252 incubation (Fig. 6a, b). However, LA+ML385 pretreatment alleviated the LA-induced increase in autophagic flux (Fig. 6c, d). In addition, the levels of nuclear Nrf2 and the mRNA expression levels of Nrf2, SOD1, SOD3, CAT, and Gpx were significantly increased after LA+NK-252 treatment (Fig. 6e, f). In contrast, the levels of nuclear Nrf2 and the mRNA expression levels of Nrf2, SOD1, SOD3, CAT, and Gpx were significantly decreased after LA+ML385 treatment (Fig. 6g, h). Thus, we confirmed that ω-6 PUFAs increase autophagy levels via activation of the antioxidant pathway.

### Identification of P62 as a Keap1-interacting protein

**Fig. 7:**
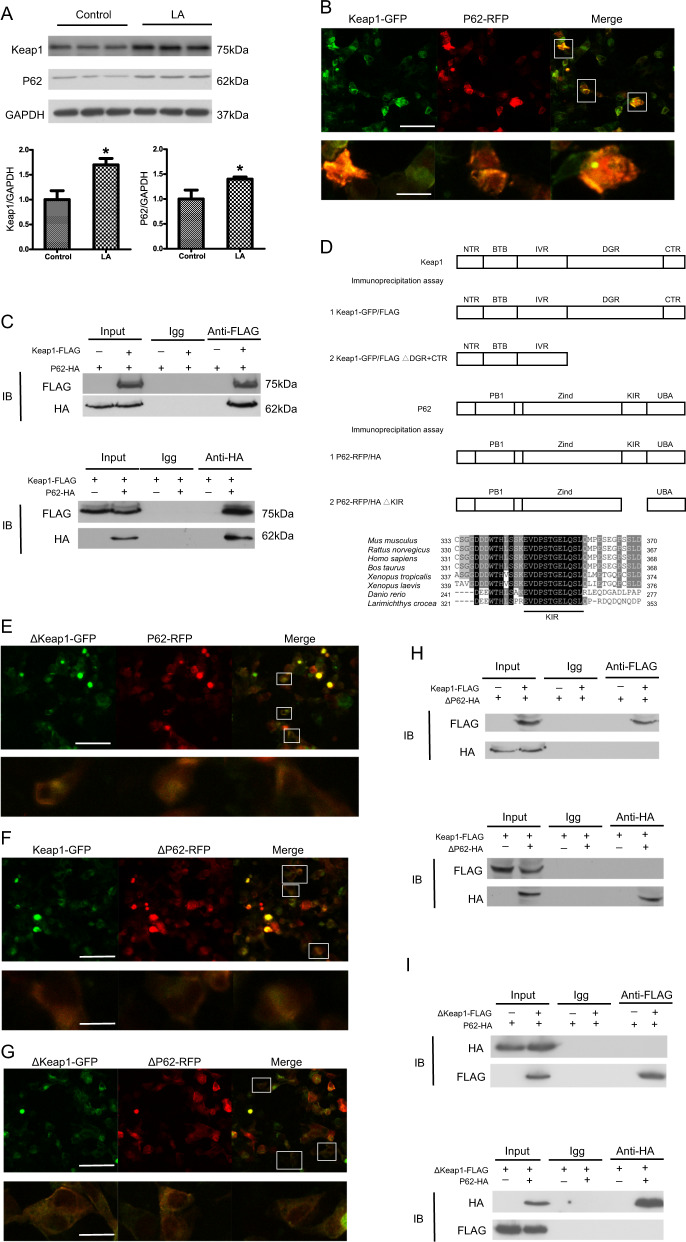
Identification of P62 as a Keap1-interacting protein. Keap1 and P62 were examined by western blot analysis and quantitated after treatment with LA (*n* = 3) (**a**). HEK293 cells were cotransfected with Keap1-GFP and P62-RFP plasmids for 12 h and then observed by confocal microscopy. Representative confocal microscopy images are shown (**b**). HEK293 cells were cotransfected with Keap1-FLAG and P62-HA for 12 h, and total cell extracts were subjected to IP using either anti-FLAG or anti-HA antibodies and an isotype control IgG. FLAG and HA in the immunoprecipitates were detected with anti-FLAG and anti-HA antibodies, respectively, by western blot analysis (**c**). Truncated mutants including ΔKeap1 and ΔP62 were constructed (**d**). Truncated mutants including ΔKeap1-GFP and ΔP62-RFP were constructed. Representative confocal microscopy images of ΔKeap1-GFP and P62-RFP, Keap1-GFP and ΔP62-RFP or ΔKeap1-GFP and ΔP62-RFP in HEK293 cells cotransfected with these different pairs of plasmids for 12 h are shown (**e**–**g**). The lysates of the two groups of HEK293 cells cotransfected with ΔKeap1-FLAG and P62-HA or Keap1-FLAG and ΔP62-HA were subjected to IP using either anti-FLAG or anti-HA antibodies and an isotype control IgG. FLAG and HA in the immunoprecipitates of the two groups were detected with anti-FLAG and anti-HA antibodies, respectively, by western blot analysis (**h**, **i**). The results are presented as the mean with SEM and were analyzed using independent *t*-tests (*P < 0.05).

Both autophagy and Keap1–Nrf2 pathways are involved in the oxidative stress response, metabolic pathways, and innate immunity^24^. Recent studies have suggested that P62, which localizes to sites of autophagosome formation, may contribute to the induction of Nrf2, but the mechanism has not been elucidated^25^. Our results indicated that the levels of Keap1 and P62 were significantly increased after LA treatment (Fig. 7a). We hypothesized that Keap1 might be associated with P62. To assess this possibility, we investigated, for the first time, whether Keap1 and P62 colocalize in the same subcellular compartments. A direct immunofluorescence assay was performed. The plasmids Keap1-GFP and P62-RFP were constructed and cotransfected into HEK293 cells. As shown by confocal microscopy, Keap1-GFP distinctly appeared to colocalize with P62-RFP (Fig. 7b). To further investigate the factors influencing colocalization, we constructed mutants of Keap1 and P62: ΔKeap1-GFP and ΔP62-RFP, respectively (Fig. 7d). Confocal microscopy revealed that deletion of the DC domains of Keap1 or the KIR domains of P62 led to the disappearance of fluorescent puncta, clearly destroying the colocalization (Fig. 7e–g). Thus, we determined that Keap1 colocalizes with P62. We then proceeded to validate if Keap1 physically interacted with P62 through coimmunoprecipitation (Co-IP) analysis. The constructed plasmids Keap1-FLAG and P62-HA were cotransfected into HEK293 cells. Western blotting showed that Keap1-FLAG coprecipitated with P62-HA (Fig. 7c). Furthermore, we performed other immunoprecipitation (IP) assays with ΔKeap1-FLAG and P62-HA or Keap1-FLAG and ΔP62-HA. The results revealed that loss of the KIR domain of P62 also abolished binding to Keap1-FLAG (Fig. 7h). Additionally, deletion of the DC domains of Keap1 eliminated binding of Keap1 to P62-HA (Fig. 7i). Taken together, our findings show that P62 interacts directly with Keap1 and causes Nrf2 to dissociate from Keap1 and translocate to the nucleus, where it activates target genes involved in the antioxidant defense.

### Nrf2 binds to the P62 promoter

**Fig. 8:**
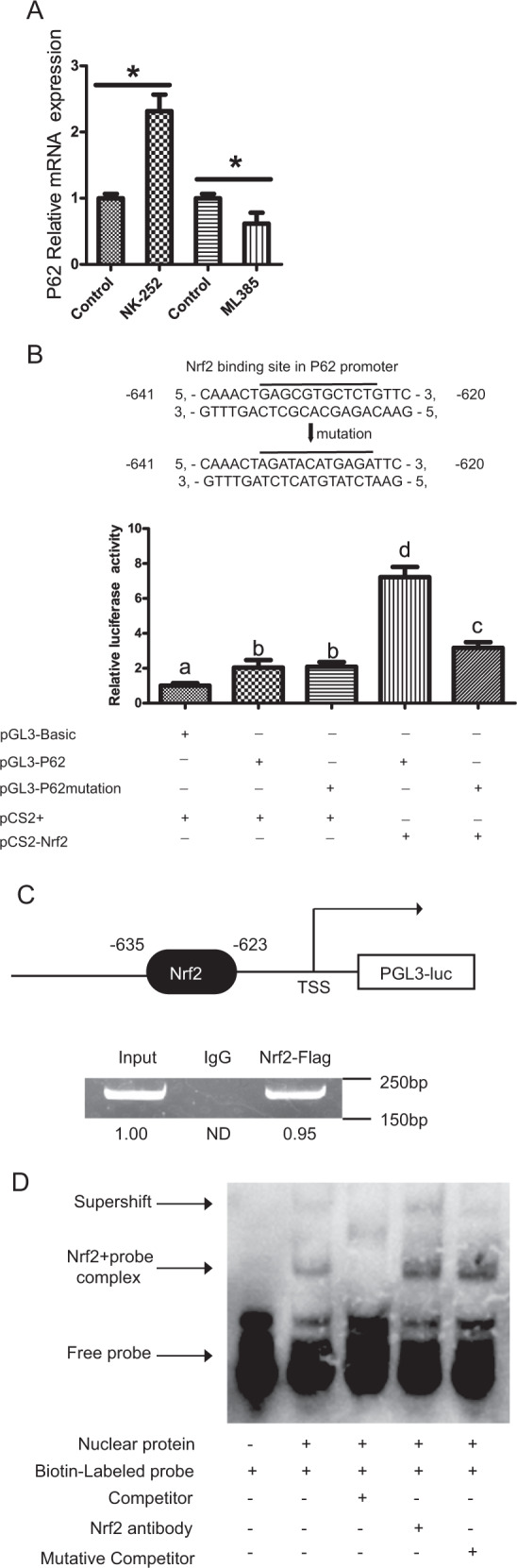
Nrf2 binds to the P62 promoter. The mRNA expression levels of P62 were analyzed after treatment with a Nrf2 pathway inhibitor (5 μM ML385) or activator (2 μM NK-252) (*n* = 6) (**a**). Relative dual-luciferase activity analysis of Nrf2 in the P62 promoter in HEK293 cells. The bars indicate the relative luciferase activity (*n* = 3). PRL-CMV and PGL3-Basic were used as the controls. The amount relative to the internal control is expressed as the mean ± SEM (*n* = 3). Significant differences compared to the control are indicated (**b**). ChIP assay identification of Nrf2 binding sites in the P62 promoter region in large yellow croaker. The band signals were quantified with densitometric software, and the input was set to 1.00 as the control (**c**). Double-stranded oligomers labeled with biotin on their 5′ ends were incubated with nuclear protein. A 100-fold excess of the competitor or mutated competitor oligomers was added for the competition or mutant competition assay, respectively (**d**). The results are presented as the mean with SEM and were analyzed using independent *t*-tests and Tukey’s test. Bars bearing the same letters are not significantly different among treatments (*P ≥ 0.05).

Transcriptional regulation is one of the most efficient methods in gene expression regulation. P62 may thus participate in a positive feedback loop to activate Nrf2, which in turn may stimulate transcription of the P62 gene. To assess this possibility, we used NK-252 and ML385 to activate and inhibit Nrf2, respectively. NK-252 pretreatment upregulated the mRNA levels of P62, but ML385 pretreatment downregulated it (Fig. 8a). To elucidate the mechanisms underlying the Nrf2-triggered transcriptional activation of autophagy, we further explored how Nrf2 modulated P62. ARE sequence in the promoter of P62 is conserved among mammalian species, and the homology of this sequence with the MAF recognition element (MARE) consensus sequence suggested that MAF protein may be recruited to the P62 ARE as a heterodimer with Nrf2. We found that the ARE was located at nucleotides −635 to −623 of the P62 promoter region in large yellow croaker (Fig. 8b). A P62 luciferase reporter system was generated to determine whether Nrf2 could transcriptionally regulate P62 promoter activity. Site mutation analysis of Nrf2 binding sites was conducted to estimate the importance of the putative ARE sequence (Fig. 8b). The results revealed that Nrf2 significantly enhanced the luciferase activity of P62 (Fig. 8b). Mutation of the ARE significantly reduced the luciferase activity of the P62 promoter, suggesting that Nrf2 transactivates P62 by binding to the ARE motif in the P62 promoter. To show that Nrf2 interacts with the P62 promoter in vivo, chromatin IP (ChIP) analyses of HEK293 cell extracts were performed using Nrf2 antibodies and polymerase chain reaction (PCR) primers targeting a 150-bp DNA fragment containing the ARE. ChIP analysis revealed that the P62 promoter fragment coprecipitated with Nrf2 antibodies (Fig. 8c), indicating that the transcription factor Nrf2 associates with the upstream regulatory region of P62. Having demonstrated that ARE motif was important for the transcription of P62, we next examined whether Nrf2 could bind to this site directly by electrophoretic mobility shift assay (EMSA). The putative ARE sequence of the P62 promoter could bind with the nuclear extract, and this binding was disrupted by unlabeled wild-type probes; however, the binding was restored by mutant probes (Fig. 8d). In addition, we observed a supershift during coincubation with the anti-Nrf2 antibodies compared to the control IgG (Fig. 8d). The above evidence suggests that Nrf2-activated autophagy occurs via binding of Nrf2 to the P62 promoter region.

## Discussion

In the present study, the transcriptomic results indicated that the autophagy-related genes were not significantly different between the FO and SO groups (Supplementary Fig. 2C). However, EM observations and immunoblot analysis demonstrated that dietary ω-6 PUFA increased autophagosome formation in vivo. These results suggested that ω-6 PUFAs did not induce autophagy at the transcriptional level. A similar expression pattern of autophagy in response to unsaturated fatty acids has also been reported in mammalian cells^26^. Besides autophagy, we also found a discrepancy in antioxidant ability between in vitro and in vivo experiment. This discrepancy, to some extent, may be due to complex fatty acid composition of the experimental diets in vivo study. The in vivo results showed that compared with fish oil group (ω-3 PUFAs), fish fed the diets with soybean oil (ω-6 PUFAs) had decreased antioxidant ability. The in vitro results showed that, compared with DHA (ω-3 PUFAs), LA (ω-6 PUFAs) also decreased antioxidant ability of primary cell in large yellow croaker in vitro. However, compared with medium (BSA), the antioxidant ability in LA group was significantly increased. The conclusions between in vivo and in vitro study were almost the same, although compared with the medium, LA increased antioxidant ability in vitro. This result is in agreement with the results of some previous studies on large yellow croaker and meagre (*Argyrosomus regius*)^27,28^. In conclusion, the findings of the present study suggested that ω-6 PUFAs can induce autophagy and increase the antioxidant system. Furthermore, from the transcriptomic evidence, KEGG analysis identified numerous unigenes that were enriched in various known immunity-related pathways, such as the mTOR signaling pathway (PI3K, eIF4E, and REDD1). These results gave us a clue that ω-6 PUFAs (LA) may regulate autophagy and antioxidation through mTOR signaling-related physiological process. Numerous previous studies have revealed the importance of the TOR protein in mediating the effects of PUFAs on autophagy and the Keap1–Nrf2 antioxidant system^17^. However, to the contrary, several stimuli have been shown to induce autophagy independent of mTOR^29^. We therefore thought it would be interesting to investigate whether TOR affects the ω-6 PUFA-triggered activation of autophagy and antioxidant ability.

To our knowledge, no findings have been published to date on the effects of ω-6 PUFAs on the regulatory mechanisms of autophagy and antioxidant ability. The in vivo experiment in the present study indicated that ω-6 PUFAs enhance AMPK activity and AMPKα phosphorylation. As far as we know, AMPK is one of the most well-recognized modulators of autophagy and antioxidant ability^30^ and increases in AMPK activity contribute to the activation of autophagy and the Keap1–Nrf2 antioxidant system^13,31^. In mammalian models, two main AMPK signaling pathway-mediated mechanisms have been identified for the induction of autophagosome formation^32^. The two mechanisms differ with regard to the participation of TOR-dependent and TOR-independent signaling pathways in autophagy and antioxidant system activation^33^. In this study, both AICAR and CC pretreatment before LA treatment (in the LA+AICAR and LA+CC groups, respectively) significantly affected the phosphorylation of AMPKα (Thr172) and ULK1 (Ser467, Ser555, Ser637, and Ser757) and the levels of nuclear Nrf2. Interestingly, the phosphorylation levels of ULK1 (Ser757), which is regulated only by the TOR signaling pathway, were changed after LA treatment. Recent studies on mammals have shown that the TOR signaling pathway is necessary and sufficient to inhibit autophagy through phosphorylation of ULK1 on Ser757, ATG13 on Ser355, and TFEB on Ser211^21,34–36^. We therefore monitored the phosphorylation status of these proteins and found that the phosphorylation of ULK1 (Ser757), ATG13 (Ser355), TFEB (Ser211) and the two TOR effectors raptor and TSC2 were changed after TOR activator and inhibitor treatment. These results suggested that TOR protein participated in the effects of ω-6 PUFAs on autophagy and antioxidant ability. Taken together, these results indicate, for the first time, that ω-6 PUFAs activate autophagy and antioxidation through the AMPK signaling pathway and the AMPK–TOR signaling pathway.

After revealing that ω-6 PUFAs could activate autophagy and antioxidation through the AMPK signaling pathway and the AMPK–TOR signaling pathway, we investigated the relationship between autophagy and antioxidant system. The results of the present study showed a synergistic feedback loop between the autophagy and antioxidation pathways through the P62 and Keap1 complex. Then, we found P62 interacted directly with Keap1 through its KIR motif, suggesting that P62 can compete with Nrf2 for binding to Keap1. This conclusion is supported by the work of Komatsu and his colleagues, who reported a direct interaction between murine P62 and Keap1^37^. This association between P62 and Keap1 leads to stabilization of Nrf2, enabling the transcription factor to induce gene expression from ARE-containing promoters. Furthermore, the P62 protein has been reported to stimulate the expression of genes containing an ARE in their promoter regions^25^. Previous studies have shown that a positive interrelationship of some sort exists between Nrf2 and P62, which influences the ARE gene battery, but the exact mechanism remains unresolved in fish^38^. Dual-luciferase reporter assays, ChIP and EMSAs revealed a direct link between Nrf2 and P62, suggesting that endogenous Nrf2 protein is recruited to putative binding sites on P62. Herein, we found that Nrf2 can induce P62 expression by binding directly to a conserved ARE in the P62 promoter, implying the existence of a positive feedback loop in this study.

In this work, we revealed, for the first time, that ω-6 PUFAs activate autophagy and antioxidation through the AMPK signaling pathway and the AMPK–TOR signaling pathway. Furthermore, we found that autophagy and antioxidant system in a synergistic feedback loop through the P62 and Keap1 complex (Supplementary Fig. 5). In consideration of malfunctioning of autophagy and antioxidation linked to a wide range of pathological conditions including neurodegenerative diseases and chronic inflammation, these results indicated us that ω-6 PUFAs could be used in diets to greatly aid in the prevention and treatment of multiple pathologies in clinical research^39^.

## Materials and methods

### Animal studies

The experimental protocols have been described by Li et al.^40^ (Supplementary Fig. 3A). In brief, large yellow croaker with similar size (mean weight 15.42 ± 0.01 g) were obtained from Fu Fa Aquatic Products Co., Ltd., Ningde, China, and reared in floating sea cages under conditions of 26 ± 2 °C, 29–32‰ salinity and 6–7 mg/L dissolved oxygen. The lipid sources were added at doses of 100 g/kg and included the following: FO as a source of ω-3 PUFAs (in the control diet) and SO as a source of ω-6 PUFAs. Details about fatty acid composition of the two diets (FO and SO) and fatty acid composition of the livers from diet-fed fish has been shown in supplemental figures (Supplementary Figs. 3B and 4A). The fish were fed each diet twice daily at 05:00 and 17:00 for 10 weeks. The investigator was blinded to the group allocation during the experiment.

### Transcriptomic analysis

For gene expression profiling, mRNA was extracted from the livers of FO and SO diet-fed large yellow croaker (six individuals in each group), mixed with a fragmentation buffer, and subjected to fragmentation. Using the fragmented mRNA as a template, cDNA was then synthesized. The short fragments were purified and resolved with EB buffer for end repair. Single adenine (A) nucleotides were then added, and the fragments were connected to adapters. Suitable fragments were selected as templates for PCR amplification. For quality control (QC), an Agilent 2100 Bioanalyzer and an ABI StepOnePlus Real-Time PCR System were used for quantification and qualification of the sample libraries, respectively. The libraries were sequenced on an Illumina HiSeq™ 2000 system. Then, the raw data were filtered to obtain clean data. The clean data were spliced to obtain a reference sequence for subsequent analysis. The open reading frame (ORF) of the sequence was predicted with TransDecoder software. Gene expression levels were estimated using FPKM values in Cufflinks software^41^. Finally, the raw data files were submitted to the National Center for Biotechnology Information (NCBI) Sequence Read Archive (SRA). The genes were assessed against various protein databases with BLASTX, including the NCBI nonredundant protein (Nr) database and Swiss-Prot database, with a cut-off *E*-value of 10^–5^. Furthermore, we used BLASTn with a cut-off *E*-value of 10^–5^ to search the genes against the NCBI nonredundant nucleotide (Nt) database. The Nr annotations and the Blast2GO program were used to perform GO annotation of the unigenes, and the KEGG Automatic Annotation Server (KAAS) was used for pathway reconstruction. All of the data have been submitted to the NCBI Gene Expression Omnibus (GEO) database.

### Enzymatic activity assays

The activity of enzymes such as CAT, SOD, and Gpx; T-AOC; and the levels of MDA were measured as previously described^18^. AMPK activity was determined with a commercial ELISA kit according to the manufacturer’s instructions (Mlbio, Shanghai, China). One unit of enzyme activity was defined as the amount of enzyme that converts 1 μmol of substrate to product per minute at 37 °C, and enzyme activity is expressed as the units per milligram of soluble protein.

### Ultrastructural observation

For EM, tissue specimens were fixed with 2.5% glutaraldehyde in 0.1 M sodium phosphate buffer (pH 7.2) for 3 h at 4 °C, washed in the same buffer for 1 h at 4 °C and postfixed with 1% osmium tetroxide in sodium phosphate buffer for 1 h at 4 °C. The tissues were then dehydrated in a graded series of ethanol starting at 50% (for 10 min per step) after two washes in propylene oxide. The tissue specimens were embedded in araldite. Ultrathin sections were stained with Mg-uranyl acetate and lead citrate for transmission electron microscopy (TEM) evaluation.

### Hepatocyte culture and treatment

A large yellow croaker hepatocyte line was obtained from the Fisheries College of JiMei University (Xiamen, Fujian, China). The hepatocytes were cultured in six-well plates at 1.5 × 10^6^ cells per well with Dulbecco’s modified Eagle’s medium (DMEM)/F12. After 2 d of culture, the hepatocytes were serum-starved with FBS-free DMEM/F12 for 2 h prior to receiving the experimental treatments. To elucidate the possible mechanism by which LA activates autophagy and antioxidation in hepatocytes, corresponding activators and inhibitors of the signaling pathways were used, including AICAR (an AMPK activator, S1802; Selleck Chemicals, Houston, TX, USA), CC (an AMPK inhibitor, S7306; Selleck Chemicals), MHY1485 (a TOR activator, B5853; APExBIO Technology, Houston, TX, USA) and RAPA (a TOR inhibitor, V900930; Sigma-Aldrich, St.Louis, MO, USA). To determine the mechanisms of autophagy and antioxidation turnover, hepatocytes were exposed to the pharmacological autophagy–lysosome pathway activator RAPA, the autophagy–lysosome pathway inhibitor 3-MA (HY-19312; Monmouth Junction, NJ, MCE) or CQ (HY-17589; MCE), the Nrf2 pathway activator NK-252 (HY-19734; MCE), or the Nrf2 pathway inhibitor ML385 (HY-100523; MCE).

### Cell viability

A CCK8 assay was performed to test cell viability according to standard protocols (Beyotime Institute of Technology, Shanghai, China). The results are expressed as the percentage cell viability, which was determined as the ratio of the optical density in the experimental wells to the optical density in the control wells.

### Quantitative real-time PCR (RT-qPCR)

RT-qPCR was performed as previously described^1^ (Supplementary Fig. 4B). Three replicate extractions were performed for each sample. The primers were designed according to published sequences (S3). To calculate the expression of genes, the comparative cycle threshold (CT) method (2^−▵▵CT^ method) was adopted, and each given value indicates the *n*-fold difference relative to the calibrator result^42^.

### Western blot analysis

Nuclear and cytoplasmic proteins were extracted from hepatocytes using a Nuclear and Cytoplasmic Protein Extraction Kit (Thermo Fisher Scientific, Waltham, MA, USA) according to the manufacturer’s instructions. The protein content was quantified using a Bradford Protein Assay Kit (Beyotime Institute of Technology). Equal amounts of protein (10 µg) were loaded into gel wells and separated by 10% sodium dodecyl sulfate polyacrylamide gel electrophoresis. The proteins in the gels were transferred to polyvinylidene fluoride (PVDF) membranes, which were then blocked at room temperature for 2 h. The PVDF membranes were incubated with primary antibodies overnight followed by secondary antibodies for 2 h and developed with enhance chemiluminescence (ECL) reagents. Antibodies against AMPKα (cat. no. 5832), phosphor-AMPKα (Thr172) (cat. no. 50081), raptor (cat. no. 2280), phosphor- raptor (Ser792) (cat. no. 2083), TSC2 (cat. no. 4308), phosphor-TSC2 (Ser1387) (cat. no. 23402), ULK1 (cat. no. 8054), phosphor-ULK1 (Ser467) (cat. no. 4634), phosphor-ULK1 (Ser555) (cat. no. 5869), phosphor-ULK1 (Ser637) (cat. no. 14205), phosphor-ULK1 (Ser757) (cat. no. 14202), ATG13 (cat. no. 13468), phosphor-ATG13 (Ser355) (cat. no. 26839), TFEB (cat. no. 37785), and phosphor-TFEB (Ser211) (cat. no. 37681) were obtained from Cell Signaling Technology (Beverly, MA, USA). Polyclonal anti-LC3 (cat. no. 7543) was obtained from Sigma-Aldrich. Nrf2 antibodies (cat. no. 62352) were obtained from Abcam (Cambridge, MA, USA). Polyclonal LaminB antibodies (cat. no. 374015) were obtained from Santa Cruz Biotechnology (Dallas, TX, USA). Anti-glyceraldehyde 3-phosphate dehydrogenase (GAPDH) antibodies (cat. no. 309154) and HRP-conjugated secondary antibodies were obtained from Golden Bridge Biotechnology (Beijing, China). All experiments were repeated at least three times. The densities of the protein bands were normalized to those of LaminB or GAPDH, which served as the internal control. All the band intensities were quantified using NIH Image 1.63 software (Rawak Software Inc., Stuttgart, Germany).

### Detection of autophagic vesicles

Cells were cultured in 24-well plates and treated with the appropriate reagents for the required periods. Thereafter, the cells were incubated with 2 μM AO (HY101879; MCE), 50 μM MDC (30432; Sigma-Aldrich) or 100 nM LysoTracker Red (C1046; Beyotime Institute of Technology) for 30 min, washed with PBS three times, and then immediately observed with a laser scanning confocal microscope (Nikon, Tokyo, Japan) to visualize the intensity of fluorescence. The fluorescence of the stained cells was quantified on a CytoFlex Flow Cytometer (Beckman Coulter, Brea, CA, USA).

### Fluorescence and confocal microscopy

Keap1-GFP and P62-RFP were constructed in our laboratory. The following truncated plasmids of Keap1-GFP and P62-RFP were constructed: ΔKeap1-GFP and ΔP62-RFP. All plasmids were confirmed by DNA sequencing. HEK293 cells were cotransfected with the Keap1-GFP or ΔKeap1-GFP and P62-RFP or ΔP62-RFP plasmids for 12 h. All assays were performed with three independent transfections. HEK293t cells cotransfected with the indicated plasmids were cultured in confocal dishes and fixed with 4% paraformaldehyde. Fluorescence was observed and analyzed with a Nikon Multizoom AZ-C2^+^ Confocal Microscope (Nikon, Tokyo, Japan).

### Co-IP

Keap1-FLAG and P62-HA were constructed in our laboratory. The following truncated plasmids of Keap1-FLAG and P62-HA were constructed: ΔKeap1-FLAG and ΔP62-HA. All plasmids were confirmed by DNA sequencing. For IP analysis, the treated cells were collected and disrupted in IP Lysis Buffer containing protease inhibitors. The total cell extracts were mixed with precleared protein G and then incubated for 3 h at 4 °C. The beads were collected by centrifugation, washed three times using washing buffer and then subjected to western blotting as described previously.

### Dual-luciferase reporter assay

The P62 promoter was constructed into the pGL3-Basic vector with a ClonExpress II One Step Cloning Kit (Vazyme Biotech, Nanjing, Jiangsu, China). The Nrf2 binding sites in the P62 promoter from large yellow croaker were predicted with JASPAR (http://jaspar.genereg.net). The site mutation construct was named P62-Mutation (AGATACATGAGA). Firefly and Renilla luciferase activity were measured using a Dual-Luciferase Reporter Assay System (Promega, Madison, WI, USA) according to the manufacturer’s instructions. Briefly, at 48 h post transfection, HEK293t cells in 24-well plates were washed twice with 100 μl of PBS and then lysed with 2 μl of 1× passive lysis buffer at room temperature for 15 min. The cell lysate (20 μl) was transferred to a plate, and 50 μl of luciferase assay reagent II and 1× Stop & Glo reagent were added in sequence. Then, firefly and Renilla luciferase activity were detected in duplicate readings using an Infinite 200 plate reader (Tecan, Männedorf, Switzerland).

### ChIP

The pGL3-P62 promoter vector and pCS2-Nrf2-flag vector were cotransfected into HEK293t cells at a ratio of 1:2. After 48 h of transfection, the HEK293 cells were fixed with 1% formaldehyde at 37 °C for 10 min. Then, a ChIP assay was performed according to the ChIP Kit (Thermo Fisher Scientific) protocol.

### EMSA

Nuclear proteins were prepared and quantified by the bicinchoninic acid method. The nuclear extracts were incubated with oligonucleotide duplexes according to the manufacturer’s instructions in the EMSA Kit (Thermo Fisher Scientific), detected by electrophoresis on 4% native polyacrylamide gels and ultimately transferred to nylon membranes. The transferred DNA was cross-linked to the membrane and detected with chemiluminescence reagents. Competition analyses were performed with a 100-fold excess of unlabeled oligonucleotide duplex with or without mutation. For the supershift assay, anti-Nrf2 antibodies or control IgG was added to the EMSA reaction mixture after the binding reaction, and the reactions were incubated at 25 °C for 30 min further before electrophoresis.

### Statistical analysis

The results are presented as the mean with SEM, and the data were evaluated by independent *t*-tests and Tukey’s test. The analyses were performed with SPSS 19.0 (IBM, Armonk, NY, USA). Bars bearing the same letters are not significantly different among treatments (*P ≥ 0.05).

## Supplementary information

Supplementary information

Supplementary fig. 1

Supplementary fig. 2

Supplementary fig. 3

Supplementary fig. 4

Supplementary fig. 5
