# Supplementary material for: ω-6 Polyunsaturated fatty acids (linoleic acid) activate both autophagy and antioxidation in a synergistic feedback loop via TOR-dependent and TOR-independent signaling pathways
Source: Cell Death Dis. 2020 Jul 30;11(7):607. doi: 10.1038/s41419-020-02750-0 (PMC7393504; doi:10.1038/s41419-020-02750-0)
Supplement: Supplementary file 1 — Supplementary information [file 41419_2020_2750_MOESM1_ESM.docx]

**Supplementary Fig. 1.** Liver transcriptome analysis of the effects of ω-6 PUFAs on autophagy and antioxidation. Differentially expressed genes (**A**) and functional annotation of the genes in large yellow croaker based on GO categorization (**B**) and KEGG categorization (**C**).

**Supplementary Fig. 2.** Statistically significant KEGG classifications of large yellow croaker genes. *P*-value < 0.05 indicated that the gene was significantly altered in fish fed soybean oil diets relative to that observed in fish fed fish oil diets (**A**). Differentially expressed genes related to immunity-relevant pathways. Identification of all differentially expressed genes was based on *P* < 0.05. *P*-value < 0.05 indicated that the gene was significantly altered in fish fed soybean oil diets relative to that observed in fish fed fish oil diets. The absolute value of “Fold-change” is the magnitude of up- or downregulation for each gene after fasting. “+” indicates upregulation, and “-” indicates downregulation (**B**). Differentially expressed genes related to autophagy pathways. Identification of all differentially expressed genes was based on *P* < 0.05. *P*-value < 0.05 indicated that the gene was significantly altered in fish fed soybean oil diets relative to that observed in fish fed fish oil diets. The absolute value of “Fold-change” is the magnitude of up- or downregulation for each gene after fasting. “+” indicates upregulation, and “-” indicates downregulation (**C**).

**Supplementary Fig. 3.** Formulation and proximate analysis of the experimental diets (% dry matter). All those ingredients were supplied by Great Seven Biotechnology Co.,Ltd, China.

^a^ FO: Fish oil group; ^b^ SO: soybean oil replacing fish oil at 100%; ^c^ Mineral premix (mg or g kg^-1^ diet): CuSO_4_·5H_2_O 10 mg; Na_2_SeO_3_ (1%) 25 mg; ZnSO_4_·H_2_O, 50 mg; CoCl_2_·6H_2_O (1%) 50 mg; MnSO_4_·H_2_O 60 mg; FeSO_4_·H_2_O 80 mg Ca (IO_3_)_2_ 180 mg; MgSO_4_·7H_2_O 1200 mg; zeolite 18.35 g; ^d^ Vitamin premix (mg or g kg-1diet): vitamin D 5 mg; vitamin K 10 mg; vitamin B12 10 mg; vitamin B6 20 mg; folic acid 20 mg; vitamin B1 25 mg; vitamin A 32 mg; vitamin B2 45 mg; pantothenic acid 60 mg; biotin 60 mg; niacin acid 200 mg; α-tocopherol 240 mg; inositol 800 mg; ascorbic acid 2000 mg; microcrystalline cellulose 16.47 g ; ^e^ Phagostimulant: Glycine/ Betaine = 1:3; ^f^ Preservative: Fumarate/ Calcium pnpionabe = 1:1 (**A**). Fatty acids composition in the experimental diets (% total fatty acid). The low-level fatty acids are not list on. “—” means not detected. SFA, saturated fatty acids; MUFA, mono-unsaturated fatty acids; n-6 PUFA, n-6 poly-unsaturated fatty acids; n-3 PUFA, n-3 poly-unsaturated fatty acids; LC-PUFA, long chain-polyunsaturated fatty acids (**B**).

**Supplementary Fig. 4.** Effect of dietary lipids on liver fatty acid composition (% total fatty acid). The low-level fatty acids are not list on. SFA, saturated fatty acids; MUFA, mono-unsaturated fatty acids; n-6 PUFA, n-6 poly-unsaturated fatty acids; n-3 PUFA, n-3 poly-unsaturated fatty acids; LC-PUFA, long chain-polyunsaturated fatty acids (**A**). Primers used in the present study. βactin: Beta-actin, Nrf2: nuclear factor erythroid 2-related factor 2, SOD: superoxide dismutase, CAT: catalase, GPx: glutathione peroxidase, Beclin1: Beclin-1, ULK1: unc-51 like autophagy activating kinase 1, ATG101: autophagy related 101, ATG13: autophagy related 13, ATG5: autophagy related 5, ATG7: autophagy related 7, ATG12: autophagy related 12, ATG4b: autophagy related 4b, LC3: microtubule associated protein 1 light chain 3, GABARA: GABARA, P62: sequestosome 1 (**B**).

**Supplementary Fig. 5.** A working model of how ω-6 PUFAs (Linoleic acid) activate both autophagy and antioxidation, which are in a synergistic relationship, *via* TOR-dependent and TOR-independent signaling pathways.
