# Supplementary figures and images for: ω-6 Polyunsaturated fatty acids (linoleic acid) activate both autophagy and antioxidation in a synergistic feedback loop via TOR-dependent and TOR-independent signaling pathways
Source: Cell Death Dis. 2020 Jul 30;11(7):607. doi: 10.1038/s41419-020-02750-0 (PMC7393504; doi:10.1038/s41419-020-02750-0)

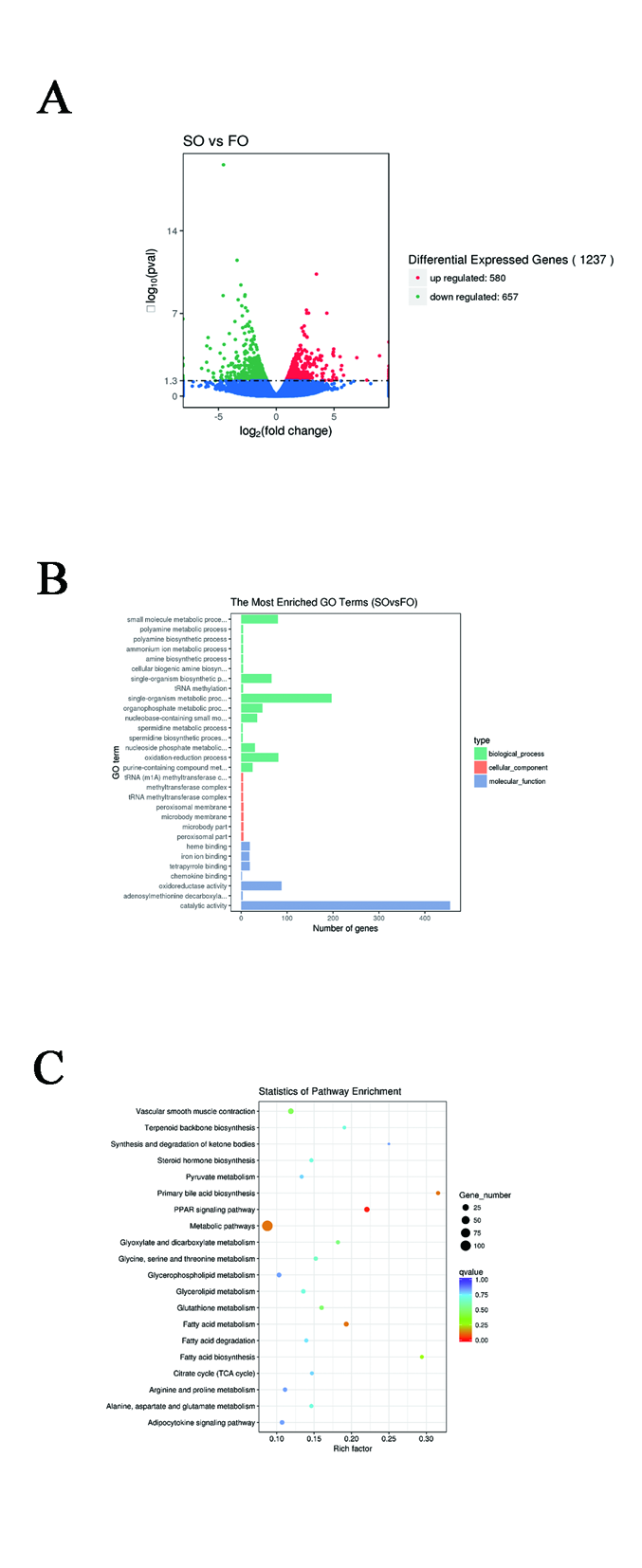

Supplement: Supplementary file 2 — Supplementary fig. 1 [file 41419_2020_2750_MOESM2_ESM.tif]

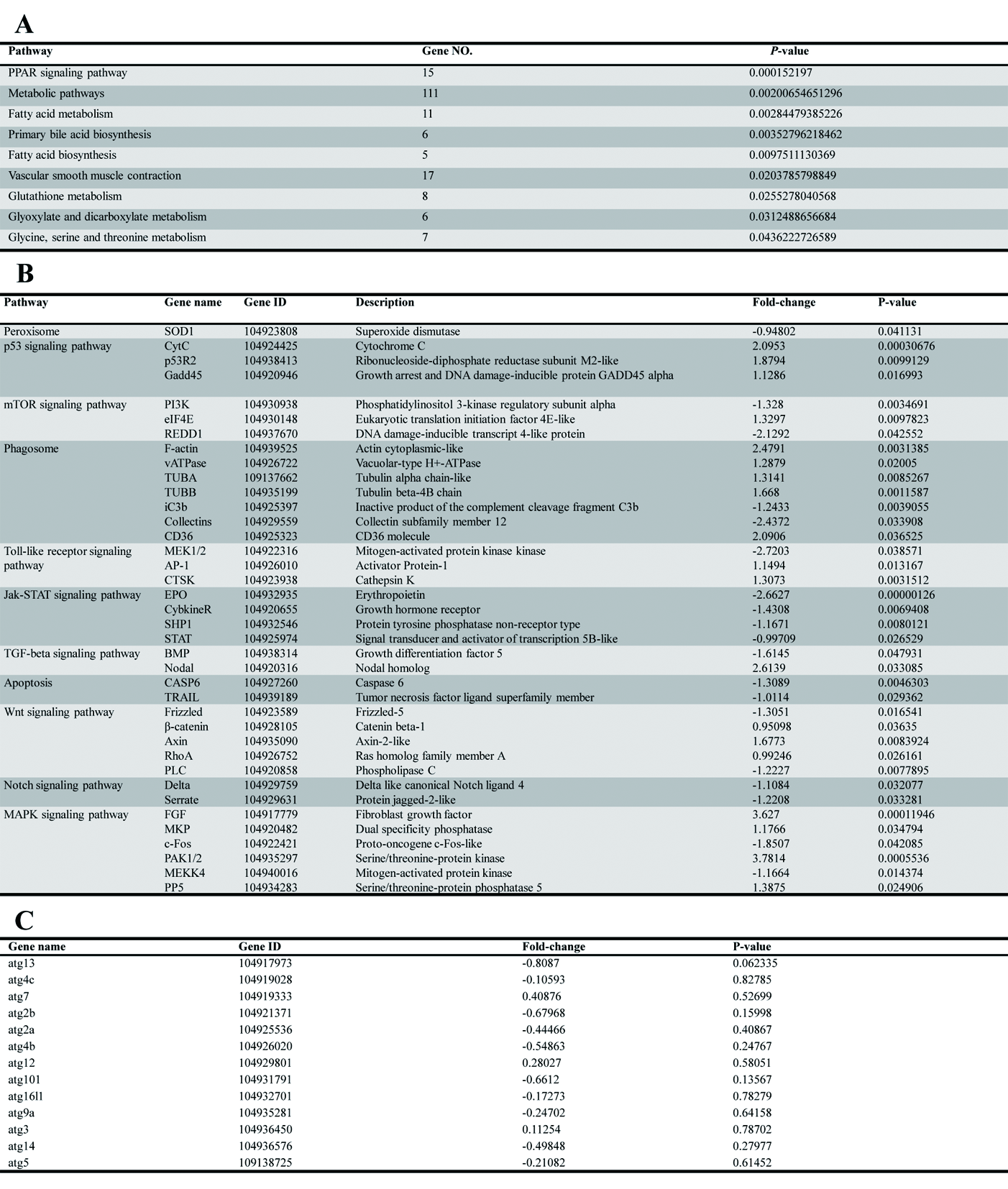

Supplement: Supplementary file 3 — Supplementary fig. 2 [file 41419_2020_2750_MOESM3_ESM.tif]

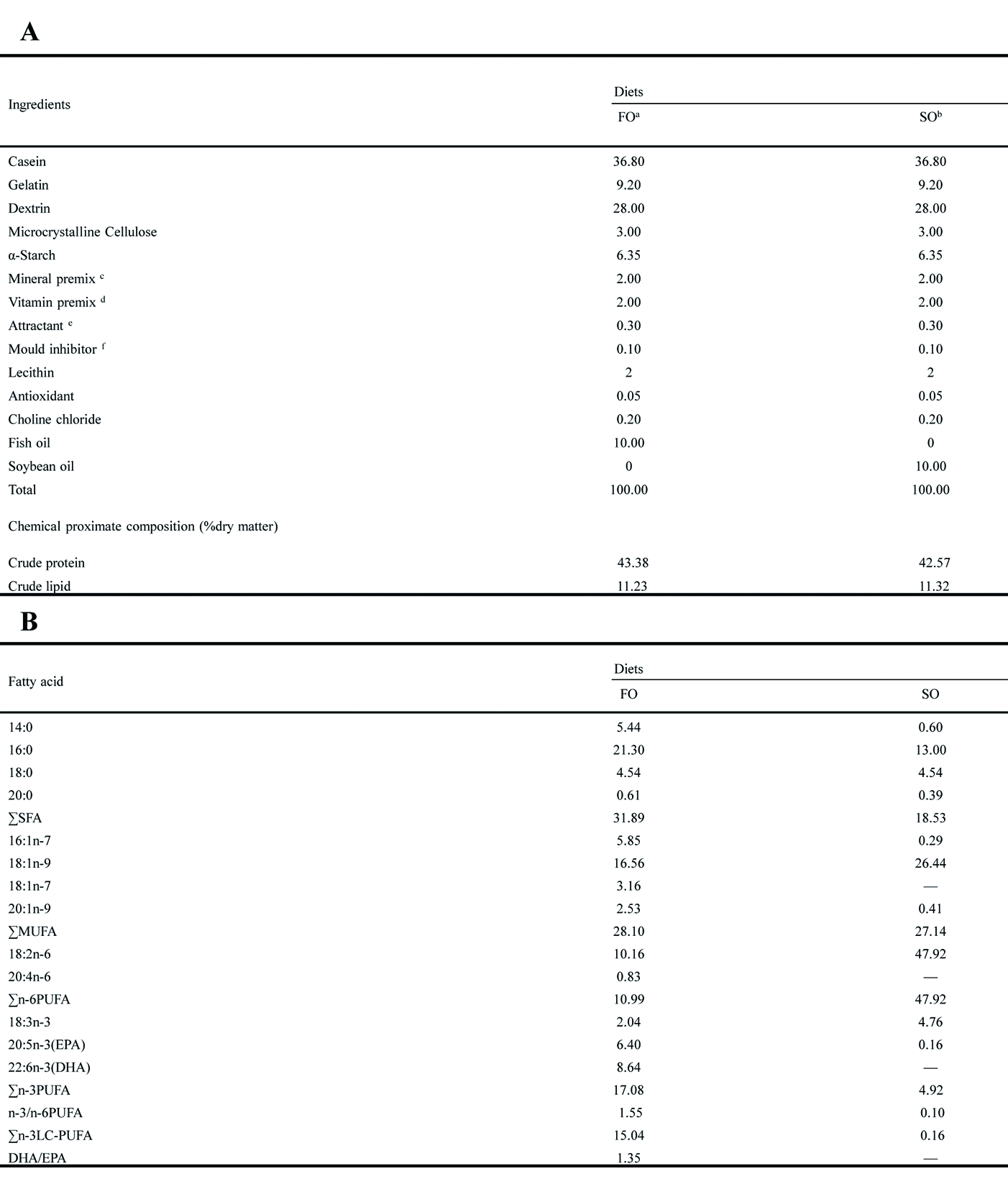

Supplement: Supplementary file 4 — Supplementary fig. 3 [file 41419_2020_2750_MOESM4_ESM.tif]

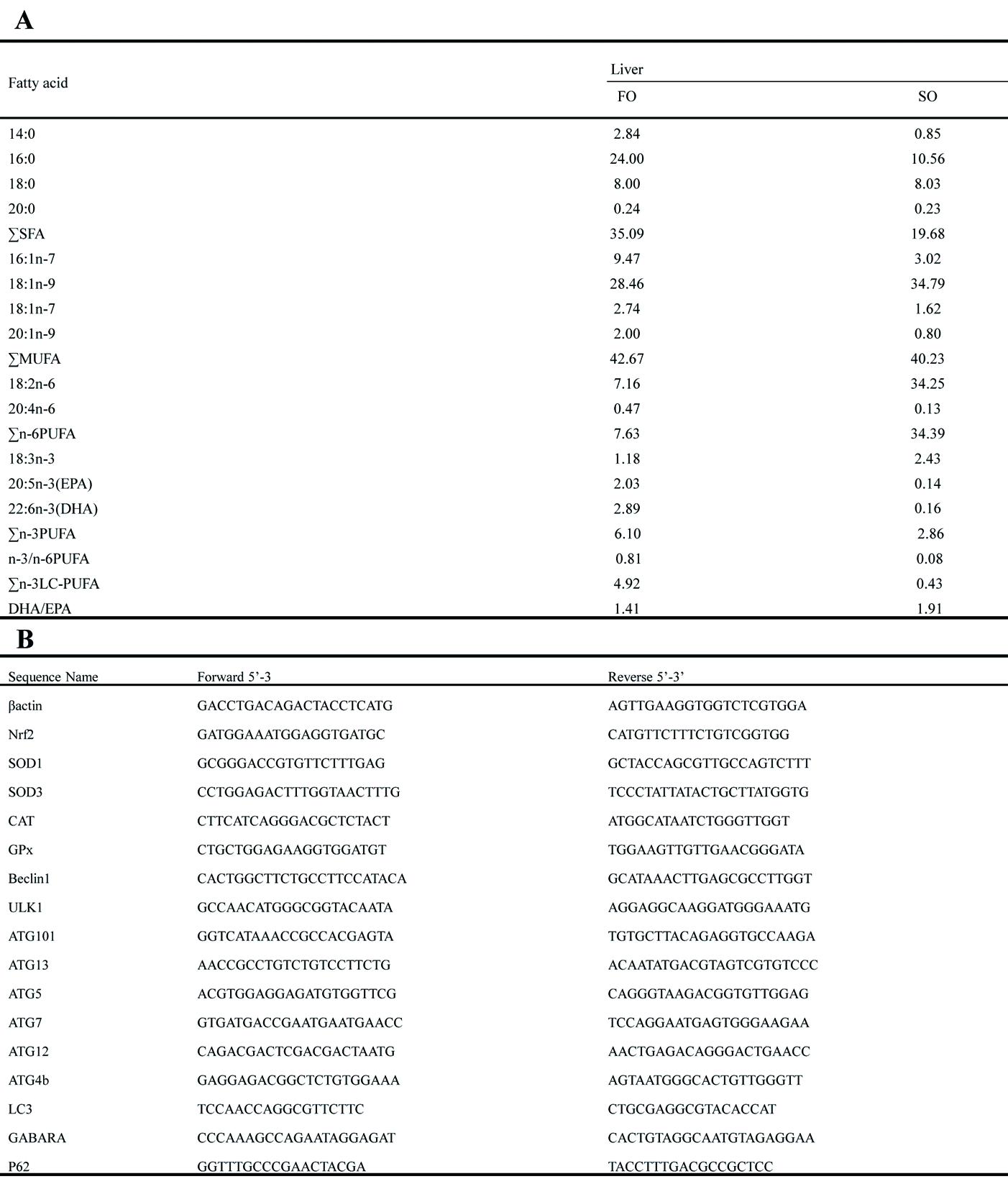

Supplement: Supplementary file 5 — Supplementary fig. 4 [file 41419_2020_2750_MOESM5_ESM.tif]

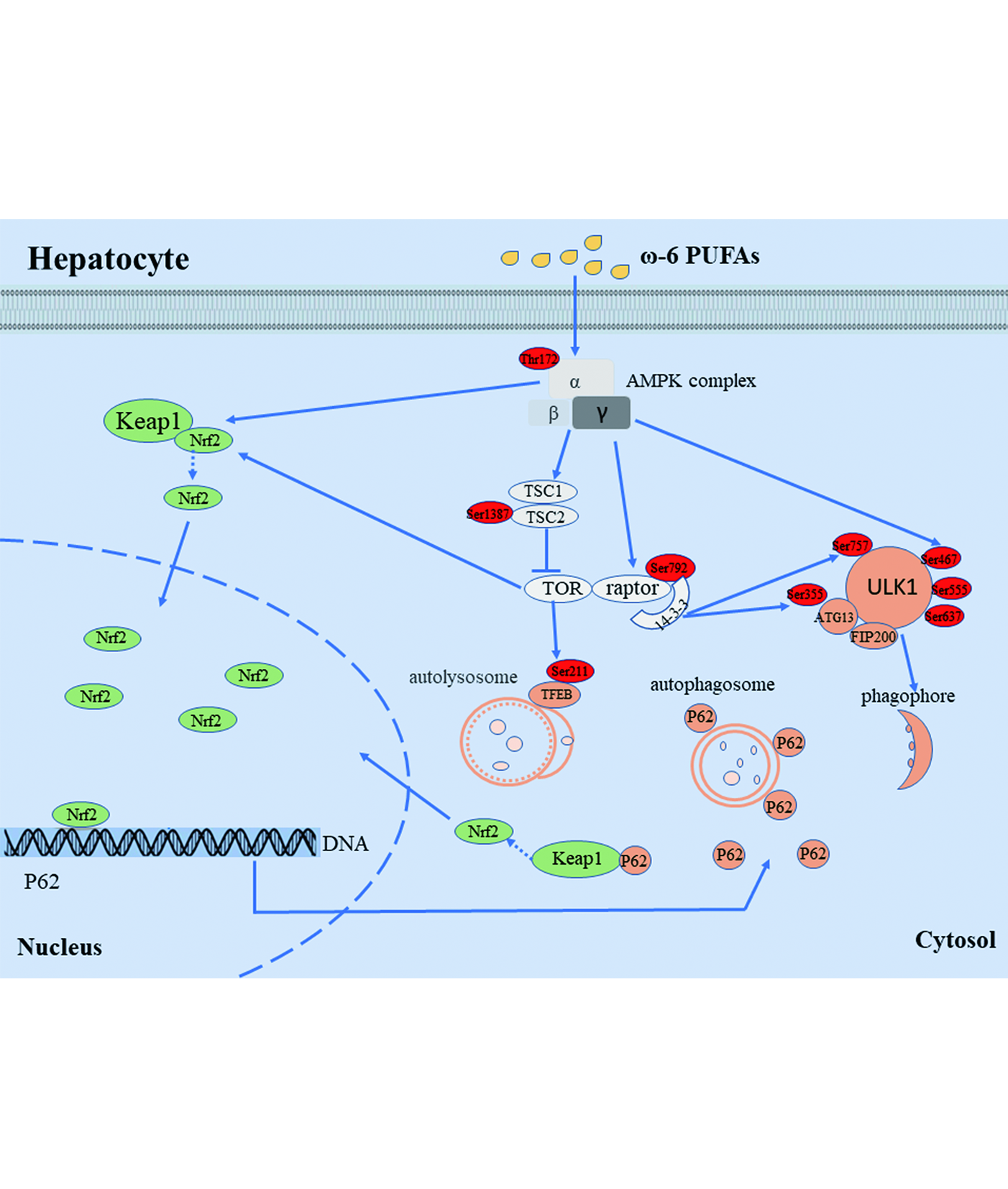

Supplement: Supplementary file 6 — Supplementary fig. 5 [file 41419_2020_2750_MOESM6_ESM.tif]
